# “Cumulative Stress”: The Effects of Maternal and Neonatal Oxidative Stress and Oxidative Stress-Inducible Genes on Programming of Atopy

**DOI:** 10.1155/2016/8651820

**Published:** 2016-07-18

**Authors:** Sara Manti, Lucia Marseglia, Gabriella D'Angelo, Caterina Cuppari, Erika Cusumano, Teresa Arrigo, Eloisa Gitto, Carmelo Salpietro

**Affiliations:** ^1^Unit of Pediatric Genetics and Immunology, Department of Pediatrics, University of Messina, 98125 Messina, Italy; ^2^Neonatal and Pediatric Intensive Care Unit, Department of Pediatrics, University of Messina, 98125 Messina, Italy

## Abstract

Although extensive epidemiological and laboratory studies have been performed to identify the environmental and immunological causes of atopy, genetic predisposition seems to be the biggest risk factor for allergic diseases. The onset of atopic diseases may be the result of heritable changes of gene expression, without any alteration in DNA sequences occurring in response to early environmental stimuli. Findings suggest that the establishment of a peculiar epigenetic pattern may also be generated by oxidative stress (OS) and perpetuated by the activation of OS-related genes. Analyzing the role of maternal and neonatal oxidative stress and oxidative stress-inducible genes, the purpose of this review was to summarize what is known about the relationship between maternal and neonatal OS-related genes and the development of atopic diseases.

## 1. Introduction

Allergic diseases including atopic dermatitis, allergic rhinitis, and asthma are some of the most common chronic diseases in the world [1]. Although extensive epidemiological and laboratory studies have been performed to identify the environmental and immunological causes of atopy, genetic predisposition seems to be the biggest risk factor for allergic diseases [2, 3]. It is known that several foetal adaptive responses to environmental factors are mediated by epigenetic changes which, impacting early-life morbidity, may exercise effects on the immune system, lung development, airway remodelling, allergen predisposition, and atopic and nonatopic inflammation, through numerous pathways [2, 4]. In particular, the onset of atopic diseases may be the result of heritable changes of gene expression, without any alteration in DNA sequences, occurring in response to early (prenatal) or later (perinatal) environmental stimuli [4]. Findings suggest that the establishment of a peculiar epigenetic pattern may also be generated by oxidative stress (OS) and perpetuated by the activation of OS-related genes [5]. Reactive oxygen species (ROS), known to be important cell-signalling molecules [6], could, in fact, set up a positive-feedback loop that induces and perpetuates atopic injury. OS, also influencing T-cell signal transduction and gene expression [7], modulates T-cell polarization toward a T helper- (Th-) 2 cellular subset [8] which might be, in turn, a further source of ROS.

OS is a specific setting also occurring in normal events such as pregnancy and birth.

Pregnancy is a physiological period associated with enhanced OS related to high metabolic turnover and elevated tissue oxygen requirements [9]. During pregnancy, increased oxygen demand augments the rate of production of ROS, and women, even during normal pregnancies, experience elevated serum OS levels [9]. Increased OS levels and reduced antioxidative capacities may contribute to the pathogenesis of perinatal [10, 11] and postnatal disorders [12, 13], such as atopic diseases [14, 15], as newborns are more prone to OS than individuals later in life [16]. Moreover, it has also been reported that OS-related maternal genetics, independently of transmission of specific alleles, may influence a child's atopic risk beginning in the uterus [17, 18].

Also during pregnancy, newborns are also continually exposed to elevated levels of ROS. At birth, newborns transit from a hypoxic intrauterine to a normoxic extrauterine environment. This increased OS further favours neonatal morbidity, also including atopy [14].

Analyzing the role of maternal and neonatal oxidative stress and oxidative stress-inducible genes, the purpose of this review was to summarize what is known about the relationship between maternal and neonatal OS-related genes and the development of atopic diseases.

## 2. Cumulative Effects of Maternal and Neonatal Oxidative Stress on the Immune System

It is well known that OS occurs early in pregnancy and continues in the postnatal period [12]. In particular, pregnancy is associated with enhanced OS related to high metabolic turnover and elevated tissue oxygen requirements [34]. On the other hand, newborns, exhibiting an accelerated production of free radicals and limited antioxidant protection, are also constitutively vulnerable to OS. Therefore, during pregnancy and intrauterine life, many factors such as hypoxia, inflammation, and infections can easily induce overproduction of free radicals (FRs) [11], exceeding the capacity of defensive mechanisms to neutralize them. The release of FRs leads to the oxidation of lipids, proteins, and polysaccharides and to DNA modifications [7–9, 19] which, in turn, increase the susceptibility of rapidly growing tissues to damage [35], as well as modulation of the immune system [10, 36].

With regard to the immune system, different immunological responses to ROS production have been reported, depending on environmental oxidative status. While normal ROS amounts have been shown to be important for T-cell function and for adequate, beneficial antimicrobial protection [37], high ROS concentrations can negatively modulate immune system responses leading to inhibited T-cell proliferation [37] and to hyporesponsivity to exogenous and/or endogenous activating stimuli [9]. In particular, OS plays a critical role as a secondary messenger in the initiation and amplification of signalling, miming antigenic effects. The antigen receptors are themselves OS-generating enzymes, contributing further to enhancing the cellular “oxidative burst” against exogenous pathogens as well as neighbouring cells [10], causing autoinflammatory and/or allergic diseases [17, 38].

Moreover, it has been suggested that OS, leading to secretion of a variety of proinflammatory cytokines and chemokines [38], elicits a polarized immune response which is closely associated with a breakdown in immune tolerance [39]. In particular, when immunoglobulin- (Ig-) E binds to specific membrane receptors, peripheral blood is activated to produce more superoxide and hydrogen peroxide (H_2_O_2_), contributing to elevated environmental OS and sterile inflammation [40] in upper and lower airways [41–43], and in the skin [44]. Furthermore, immune cells, because of higher production of ROS, are themselves particularly sensitive to OS, creating a vicious circle for the production of proinflammatory mediators and supporting a prooxidant status [9]. The activation of both the redox-sensitive transcription factor nuclear factor-kappa B (NF-*κ*B) and activator protein- (AP-) 1 and the release of proinflammatory proteins involved in immune response (e.g., interleukin- (IL-) 1, IL-6, tumour necrosis factor- (TNF-) *α*, and interferon- (INF-) *α*, as well as H_2_O_2_) are critical events in immunity, promoting stimulus-specific genes expression [17, 38]. These findings confirm the evidence that foetal immune response is prenatally influenced [45] and that the activation of maternal and neonatal OS-inducible genes may influence a child's atopic risk, early in the uterus [46, 47] (Tables 1, 2, and 3).

## 3. Epigenetic Effects on Atopic Predisposition

Epigenetics refers to information that is heritable through cell division. Epigenetic mechanisms include DNA methylation, chromatin remodelling and noncoding RNA, histone variations, and posttranslational histone modifications [48]. Epigenetic alterations can occur prenatally, perinatally, and later in life during developmental stages, with unique susceptibility to the effects of environmental exposures [48]. Uterine life is the most critical time in developmental programming; when negative environmental exposures occur, the foetal structure and its functions are irreversibly modified and subjects can be predisposed to several diseases, including allergy [49]. T-cellular differentiation into Th1, Th2, Th17, and Treg is influenced by changes in DNA/histone methylation and/or histone acetylation in naive T-cells and in cytokine promoter regions. Thus, the well-known correlation between epigenetic modifications and Th lineage has led to hypothesize that triggers inhibit Th1 and T regulatory cell differentiation, promote Th2-response, and could favour the risk of atopic predisposition [50]. Although the mechanism of this process is not fully understood, environmental changes, such as microbial burden [51], dietary changes [52, 53], and environmental pollutants [54], appear essential to initiate the cascade of epigenetic modifications that stabilize Th2 gene expression [55]. It is also likely that effects of environmental triggers are also mediated by oxidative stress which, by NF-*κ*B-induced expression of proinflammatory cytokines and methylation-mediated changing, can induce histone modifications and chromatin remodelling of proinflammatory genes, exercising further implications on foetal immune programming, atopic predisposition, and increased IgE production following allergen sensitization [49, 56].

## 4. Cumulative Effects of Oxidative Stress-Inducible Genes on the Immune System

Genetic linkage and transmission alleles analyses have highlighted the important role of oxidative stress-inducible genes on the neonatal immune system response [26, 57]. In particular, the concurrent presence of higher ROS levels and antigenic exposure has been reported to alter the methylation of T helper genes [58]. All these changes impair the differentiation of T helper cells, increasing the risk of allergic sensitization [58]. More recently, changes in the expression of small noncoding regulator microRNAs have also been suggested as being critical for mediation of imbalanced responses to allergens [59]. However, to date, it is still unclear what genes and pathways are active during pregnancy and/or at birth and which systems are down- and/or upregulated in response to perinatal OS.

There is increasing evidence that ROS, also at physiologic concentrations, might, acting as cell-signalling mediators and promoting a shift toward a Th2-skewed immune response [17, 38], play additional roles in the onset of allergic disorders [17, 38].

The lung, due to its anatomy, provides an extensive surface area available to interact with all sources of reactive O_2_ species, and a large variety of lung diseases, including allergic asthma, may be induced by ROS [26, 43]. In particular, pulmonary epithelial cells of alveolar structure appear to be the principle target for oxidant injury which, inhibiting cellular cycle progression, promotes a delayed reepithelialization process and irreversible cellular damage [60]. Moreover, airway inflammatory cells, such as macrophages [61], eosinophils, and peripheral blood monocytes [40], are themselves a likely source of ROS production [62]. Confirming these findings, studies have shown higher H_2_O_2_, nitric oxide, and superoxide levels in exhaled gases from asthmatic patients than from control subjects [63–66]. A prooxidant status also induces a wide range of biological and molecular damage in the lung. Increased release of isoprostanes and ethane, both in epithelial and in endothelial cell membranes, as well as diminished activity of proteins, such as *α*1-protease inhibitor, ascorbate, *α*-tocopherol, and superoxide dismutase (SOD), has been reported [67].

Acting on other targets, such as airway smooth muscle, inducing acetylcholine-mediated contraction [68], mucin secretion [69], and nitric oxide- (NO-) mediated neurogenic inflammation [69], ROS can also impair broncho- and vasoregulation [70, 71].

Finally, large-scale genome-wide association studies (GWAS) have demonstrated that genetic susceptibility to allergic asthma is also determined by complex interactions between genes involved in OS, such as glutamate cysteine ligase (GCLM), glutathione peroxidase (GPX1), catalase (CAT), myeloperoxidase (MPO), NADPH oxidase (CYBA, p22phox subunit), NAD(P)H, quinone oxidoreductase type 1 (NQO1), and microsomal epoxide hydrolase (EPHX1) [26] (Table 1).

As the primary cell of interface between internal and external environments, nasal mucosal epithelial cells are known to initiate the release of a cascade of proinflammatory mediators through redox pathways [20]. Moreover, these cells also exhibit the capacity to upregulate an effective antioxidant defence [20]. However, natural allergen exposure agents show the ability to interfere with oxidant/antioxidant balance, enhancing OS and upper airway inflammation [72].

Although it has been hypothesized that the role of OS in allergic rhinitis is similar to that of asthma, the exact underlying mechanism is still not understood. However, it has been reported that OS, playing a critical role in allergic asthma, can also contribute to the onset of allergic rhinitis and to enhancing the asthma-rhinitis link, as expression of united airways disease [73].

It has been widely assessed that the loss of antioxidant activities characterizes patients affected by allergic rhinitis. Studies reported that decreased activities of both antioxidant enzyme paraoxonase (PON1) [74] and reduced glutathione [20] are inversely correlated to plasma total oxidant status and to severity of disease [20]. Consequently, increased nasal fraction of exhaled NO (FENO), 8-isoprostane, leukotriene- (LT-) B4, and PGE2 levels was detected in patients with allergic rhinitis [75]. An impaired function and distribution of superoxide anion, NADPH oxidase (NOX)1, and NOX4 in allergic nasal rhinitis has also been noted, as further confirmation of the possible influence of OS on the development of allergic rhinitis [76] (Table 2).

The ability to interfere with the immune system allows ROS to induce and perpetuate skin injury, also in atopic dermatitis. In particular, authors reported that ROS, acting mainly on keratinocytes and partially on lymphocytes [77], induce oxidative protein damage in the stratum corneum, leading to the disruption of barrier functions and the exacerbation of atopic dermatitis [78]. Therefore, in response to a variety of oxidant reactants, the skin upregulates transactivating AP-1 components such as Fos and Jun, whereas it downregulates anti-inflammatory components [79]. Precisely, it has been suggested that upregulation of AP-1 may be associated with a defect in ceramide generation which could result in enhanced protein kinase-C activation, leading to excessive release of proinflammatory cytokines by keratinocytes [79]. Generally, peroxisome proliferator-activated receptors (PPARs), a member of the nuclear factor family, also influence the biological activity of keratinocytes. To be precise, PPAR isoform-*α* (PPAR-*α*) counteracts the inflammatory response by inhibition of the expression of proinflammatory genes, as well as cytokines and metalloproteases. PPAR-*α* activation also induces antioxidant enzymes (catalase, SOD) which would reduce oxidative damage and inflammatory response [21].

The oxidant/antioxidant balance is also altered in atopic dermatitis. ROS reduce the physiological antioxidant levels of a number of compounds, such as *α*-tocopherol (VE), ubiquinol-10 (CoQH2-10), ascorbic acid (VC), and glutathione (GSH), in the epidermis and dermis and thus impair the cellular redox system [80]. Evidence of enhanced protein and lipid-oxidative damage was also found in atopic dermatitis patients, as demonstrated by the increase of carbonyl moieties both in lesional and in nonlesional skin, along with higher activity of SOD, an effective scavenger of ROS [81]. Recent experimental studies support a role for oxidative/antioxidative imbalance also in the shift toward a Th2-skewed immune response, probably NO-mediated [38]. Accordingly, the administration of antioxidants to human T-cells culture downregulated Th2 polarization, with a decrease in the expression of IL-4 and IL-5, and simultaneous skewing toward a Th1- phenotype [38]. Finally, data suggest epigenetic changes linked to the development of atopic dermatitis through OS-mediated immune dysregulation [82] (Table 3).

## 5. Conclusions

To date, the exact underlying mechanisms of atopic disease are still not understood. Recently, more attention has been given to the critical role of OS-inducible genes in the pathogenesis of atopic diseases. However, in spite of much evidence linking atopic predisposition, inflammatory status, and maternal and neonatal OS, much more remains to be investigated. Moreover, a genomic approach would clarify the role of oxidant/antioxidant pathways, in order to better understand the pathogenesis of atopic diseases and identify innovative therapeutic strategies.

## Figures and Tables

**Table 1:** Oxidative stress-inducible genes and allergic asthma.

Gene	Clinical relevance	References
Glutathione S-transferases M1 (GSTM1) and P1 (GSTP1)	GSTs conjugate endogenous byproducts of OS with glutathione, enabling rapid elimination and thus defending tissues against oxidant damage; common polymorphisms exist in genes coding for various GSTs including glutathione S-transferases M1 (GSTM1) and P1 (GSTP1)	[19]

Antioxidant defence enzymes (ADE) Glutamate cysteine ligase (GCLM) Glutathione peroxidase (GPX1) Myeloperoxidase (MPO) NADPH oxidase (CYBA, p22phox subunit) NAD(P)H: quinone oxidoreductase type 1 (NQO1) Microsomal epoxide hydrolase (EPHX1) Glutamate cysteine ligase (GCLM)	They are associated with allergic and nonallergic asthma, inducing increased oxidative stress status	[11, 20, 21]

Tumor necrosis factor G-308A	It may have a protective role in asthma pathogenesis, depending on airway oxidative stress levels	[22]

Methylenetetrahydrofolate reductase (MTHFR) ORM1-like 3 (ORMDL3) Gasdermin A and B (GSDM)	In addition to foetal smoke exposure, it seems to be associated with lower airway responsiveness, lung function, and increased risk of transient wheezing, a phenotype of childhood asthma	[23] [24] [25]

Antioxidant enzyme paraoxonase (PON1)	It is inversely correlated to plasma total oxidant status and to severity of asthma	[26]

Nuclear factor (NF), erythroid-derived 2-related factor 2 (NRF2)	It has been found to be a critical regulator in protecting cells and tissues under highly oxidative microenvironments, including airways that interface with the external environment and are exposed to pollutants and other oxidant stressors	[27]

Toll-like receptor 4 (Tlr4)	It is associated with O_3_-induced lung inflammation and increased airway hyperpermeability	[28]

Heme oxygenase-1 (HMOX-1)	In addition to ozone exposure, it is responsible for the onset of allergic asthma	[29]

Transforming growth factor- (TGF-) beta1 C-509T polymorphism	This genotype is associated with an increased risk of asthma in addition to maternal smoking exposure in the uterus or to traffic-related emissions	[30]

Arginases (*ARG1 *and *ARG2*)	It may play an important role in asthma pathogenesis through effects on nitrosative stress	[31]

**Table 2:** Oxidative stress-inducible genes and allergic rhinitis.

Gene	Clinical relevance	References
Glutathione S-transferases- (GSTs-) 1 polymorphism	It may exert protective effects in allergic rhinitis, decreasing oxidative stress status	[19]

Tumour necrosis factor (TNF) rs1800629 Toll-like receptor 4 (Tlr4) rs1927911	They are associated with a higher risk of allergic rhinitis	[22, 28]

**Table 3:** Oxidative stress-inducible genes and atopic dermatitis.

Gene	Clinical relevance	References
Glutathione S-transferases- (GSTs-) 1 polymorphism	It is associated with atopic dermatitis susceptibility in a Korean population	[19]

MicroRNA-223 or hypomethylation of the thymic stromal lymphopoietin (TSLP) gene 59-CpG island (CGI)	It predisposes the host to development of atopic dermatitis when combined with exposure to oxidative stress	[32]

Tumour necrosis factor (TNF) promoter region (TNF-a-308G/A) and linked	It is linked to oxidative stress-mediated atopic dermatitis	[22]

Nitric oxide polymorphism (T276 (276C/T, nNOS) + C186 (-186A/C, nNOS) + X (CCTTT), nNOS + G954 (-954G/C, iNOS) +220 (TAAA), niNOS + G894 (894C/G, eNOS) + a (VNTR), eNOs)	It is related to clinical and functional manifestations of bronchial asthma and atopic dermatitis	[33]
